# Maternal Factors and Utilization of the Antenatal Care Services during Pregnancy Associated with Low Birth Weight in Rural Nepal: Analyses of the Antenatal Care and Birth Weight Records of the MATRI-SUMAN Trial

**DOI:** 10.3390/ijerph15112450

**Published:** 2018-11-03

**Authors:** Dilaram Acharya, Jitendra Kumar Singh, Rajendra Kadel, Seok-Ju Yoo, Ji-Hyuk Park, Kwan Lee

**Affiliations:** 1 Department of Preventive Medicine, College of Medicine, Dongguk University, Gyeongju 38066, Korea; dilaramacharya123@gmail.com (D.A.); medhippo@hanmail.net (S.-J.Y.); skeyd@naver.com (J.-H.P.); 2 Department of Community Medicine and Public Health, Janaki Medical College, Tribhuvan University, Janakpur 44618, Nepal; jsingdj@gmail.com; 3 Personal Social Services Research Unit, London School of Economics and Political Science, London, WC2A2AE, UK; r.kadel@lse.ac.uk

**Keywords:** antenatal care services, low birth weight, maternal factors, Nepal

## Abstract

Low birth weight (LBW) remains a major public health problem in developing countries, including Nepal. This study was undertaken to examine the association between LBW and maternal factors and antenatal care service utilization, in rural Nepal, using data obtained for a capacity-building and text-messaging intervention, designed to enhance maternal and child health service utilization among pregnant women, in rural Nepal (“MATRI-SUMAN”). The study used a clustered randomized controlled design and was conducted during 2015–2016. We investigated maternal and antenatal care service utilization determinants of LBW, using a logistic regression model. Of the four hundred and two singleton babies, included in the present study, seventy-eight (19.4%) had an LBW (mean (SD), 2210.64 (212.47)) grams. It was found that Dalit caste/ethnicity, illiteracy, manual labor, a female baby, and having more than four family members were significantly positively associated with LBW. In addition, mothers who did not visit an antenatal care (ANC) unit, visited an ANC < 4 times, did not take iron and folic acid (IFA), de-worming tablets, and mothers that did not consume additional food, during pregnancy, were more likely to have an LBW baby, than their counterparts. The MATRI-SUMAN intervention and availability of a kitchen garden at home, were found to reduce the risk of LBW. Nepalese child survival policies and programs should pay attention to these maternal and antenatal care service utilization factors, while designating preventive strategies to improve child health outcomes.

## 1. Introduction

Low birth weight (LBW) has been defined as a birth weight <2500 grams [1]. In 2013, an estimated 16 per cent (22 million) of all babies born globally, had LBW and 96% of these babies were born in developing countries [1,2]. In Nepal, a recent study, using the Nepal Demographic and Health Survey 2011 data showed that the prevalence of LBW was 15.4% [3]. It was noted that LBW babies are at a greater risk of dying in the first year of life [4]. LBW can result from either intra-uterine growth restriction, small-for-gestational-age (born before 37 weeks of gestation) or a combination of the two [1,5,6]. The majority of LBW cases in developing countries are due to intra-uterine growth retardation, while pre-term birth is common in the developed countries [7]. In addition to adverse consequences, such as, increased neonatal morbidity and mortality, inhibition of growth and cognitive development, and an increased risk of chronic disease development, later in life, LBW also has substantial cost burdens on health care systems and society [1,8,9,10]. Several recent studies have concluded that LBW increases the risk of non-communicable diseases, such as, diabetes and cardiovascular conditions, later in life [11,12,13]. Moreover, LBW has been reported to be associated with negative effects on long-term cognitive and motor development, and on decision-making [14].

Numerous direct and indirect factors have been associated with LBW [15,16,17]. Socio-demographic and economic characteristics, age, household food security, and the use of maternity services are some of the indirect determinants identified, whereas race, maternal height and pre-pregnancy weight, gestational weight gains, calorie intake during pregnancy, perinatal morbidity, parity, infant’s sex, alcohol and cigarette consumption during pregnancy, and prior history of prematurity, among others, have been reported to be direct determinants [16,17,18,19]. 

Few community-based studies have assessed the risk factors of LBW in Nepal. In a recent Nepalese study on nationally representative samples, it was reported that 12% of infants had an LBW, and that mothers who did not attend antenatal care units, did not take iron tablets during pregnancy, and resided in the westernmost region of the country were at a significant risk of having an LBW baby [20]. Given that the majority (65%) of births took place at home [21] and no significant change was detected between the percentages of LBW babies observed in the 2011 and the 2016 Demographic and Health Surveys (~12% in both surveys) [22], LBW is now considered an important public health problem. Therefore, further understanding of the risk factors of LBW is required to support the early identification of those at risk and facilitate the implementations of evidence-specific interventions to reduce the long-standing problem of LBW in Nepal. To fulfill the existing evidence gap, we aimed to examine the association between low birth weight and maternal factors and utilization of antenatal care services, in rural Nepal.

## 2. Materials and Methods

### 2.1. Study Design, Population, and Sampling

We used data from a capacity-building and text-messaging intervention that was developed to enhance maternal and child health service utilization among pregnant women, in rural Nepal (“MATRI-SUMAN”), which was conducted using a clustered randomized controlled design. Pregnant women aged 15–49 years, in their second trimester (13–28 weeks of gestation), from fifty-two clusters (wards) of six village development committees (VDCs), from the Dhanusha district, were selected for the MATRI-SUMAN trial, using multistage cluster sampling method. During the first stage of the MATRI-SUMAN trial, two healthcare facilities were selected as a primary sampling unit. In the second stage, six of the twelve VDCs, in the catchment areas of these two healthcare facilities, were selected, using a stratified random sampling method. In the intervention group, Female Community Health Volunteers (FCHVs) were trained in capacity development skills through reinforcement training, supervision, and monitoring of maternal and child health care services. Pregnant women received periodic health promotion texts about maternal and child health (MCH) components, via a mobile messaging service, while participants in the control group received the usual care. Details of the study design have been reported in a previously published paper [23]. 

**Figure 1:**
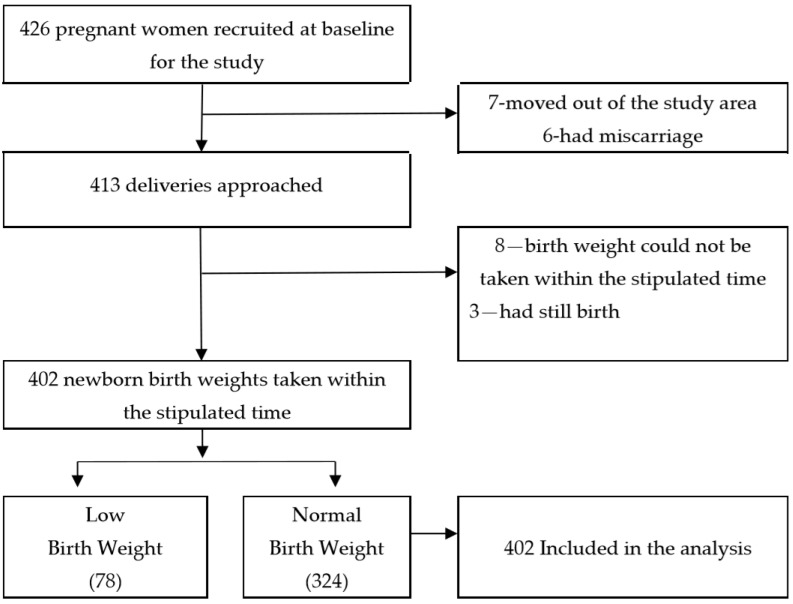
Flow chart showing the process of sample selection.

A total of four hundred and twenty-six pregnant women aged 15–49 participated in the MATRI-SUMAN trial. Of the four hundred and twenty-six participants interviewed at baseline, four hundred and thirteen of them were approached at postnatal follow-up and the remainder were not available for follow-up (of the thirteen missing cases, seven had moved to their parental home and six had a miscarriage). Additionally, we were not able to measure the birth weights, within the stipulated time, for eight cases and three mothers had still births, giving us a sample of four hundred and two mothers with live birth babies. Among the four hundred and two cases, seventy-eight newborn babies had low birth weight and the remaining three hundred and twenty-four babies had normal birth weight. We included four hundred and two live birth cases for the statistical analyses (Figure 1). The criteria we used for data selection were as follows; singleton live birth with birth weight measured within one hour of birth, for institutional delivery, and within forty-eight hours of birth, for home delivery. A pan balance was used to measure birth weights in a health facility (hospital/birthing center) and a color-coded spring balance was used by a local FCHV, for the home deliveries. Three sets of validated questionnaires that were adopted from the Nepal Demographic and Population Health Survey, 2011, were used to collect information [21]. These questionnaires addressed; (i) the socio-demographic and household characteristics of pregnant women, (ii) obstetric history of pregnant women, and iii) newborn information. The study data contained information on maternal factors, newborn weight, and maternal utilization of antenatal care services. 

### 2.2. Definition of Variables

A low birth weight (LBW) was defined as one <2500 grams. Birth weights were classified as LBW (<2500 grams) or (ii) normal (≥2500 grams or more), and birth weight was the dependent variable in the present study. Independent variables examined were chosen, as previously described [24]. Age was classified as: (i) <20 years, (ii) 20–34 years, or (iii) ≥35 years. Ethnicity was classified as, (i) upper caste group—Brahmin, Chhetri, and other relatively-advantaged Terai caste groups (Yadav, Shaha, Thakur), (ii) Adibasi/Janajati– Janajati or indigenous groups, and (iii) Dalit (relatively disadvantaged) [25]. Education was recorded as number of completed years of education and classified as, (i) illiterate—no education, (ii) primary—1 to 5 years of education, and (iii) secondary and above (≥6 years of education) [24]. Occupations were categorized as, (i) business or private/government work or involved in household work; (ii) agricultural work on own farms, and (iii) manual labor. Incomes were classified using monthly family incomes and categorized using terciles, (i) 1st tercile (income <14,333 Nepalese Rupees/month (NRs/month), (ii) 2nd tercile (14334–23666 NRs/month), of (iii) 3rd tercile (>23666 NRs/month). Parity was categorized as primiparous or multiparous. Head of household was included as an indicator of maternal autonomy and classified as, (i) the pregnant women herself or (ii) husband/mother-in-law, father-in-law, or others. Personal dietary habits were recorded as vegetarian or non-vegetarian. Family size was classified as ≤4 or >4. Number of living rooms were classified as, (i) sufficient if there were not >2 family members sleeping per room or (ii) insufficient if >2 family members slept in a room. Reponses regarding the presence of domestic animals in a household and of a home kitchen garden were recorded as “yes” or “no”. Antenatal care (ANC) visits were categorized as, (i) no ANC visit, (ii) <4 ANC visits, and (iii) ≥4 ANC visits. Iron and folic acid (IFA) consumption and tetanus and diphtheria (TD) immunization were recorded as “yes” or “no”. Similarly, other components of antenatal care (like de-worming, adequate rest, and sleep (Eight hours of sleep during night time and two hours during day time), additional food (defined as one extra meal per day as recommended by the government of Nepal) were also coded as “yes” or “no”. 

### 2.3. Statistical Analysis 

The Chi-square test (unadjusted) was used to examine the association between the LBW and the categorical independent variables of interest. All factors, significant by the unadjusted analysis, were included in the multiple logistic regression analysis to control for confounding effects. Unadjusted and adjusted odds ratios, with their 95% confidence intervals (CI), were reported. Variables having *p*-value ≤0.1 were entered in a final multivariate logistic regression model with a backward elimination and the analysis results with a *p*-value of <0.05 was considered statistically significant. Statistical analysis was performed using the Statistical Package for Social Sciences version 21.0 (SPSS, IBM, Armonk, NY, USA).

### 2.4. Ethical Considerations

The study protocol was approved by the Nepal Health Research Council (approval no: 101), the Ethics Committee of the Institute of Medical Sciences, Banaras Hindu University, India (approval no: ECR/ 526/Inst/UP/2014 Dt.31.1.14) and District Public Health Office, Dhanusha, Nepal (Ref. 2245). Additional ethical approval for the data analysis was obtained from the Institutional Review Board of Janaki Medical College, Nepal. The study objectives and procedures were explained, and a written informed consent was taken from each participant, before the data collection process began. Personal identifiers were removed before data analysis.

## 3. Results

### 3.1. Status of Birth Weight

**Figure 2:**
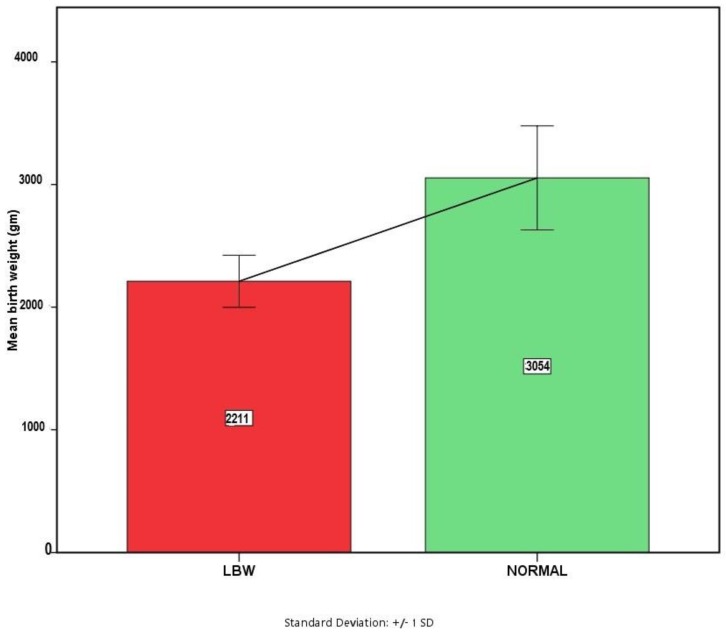
Status of birth weights among newborns.

Figure 2 shows the distribution of average birth weight (with standard deviations) of the newborn babies. The average birth weight for the LBW babies was 2210.64 grams with a standard deviation of 212.47 grams, whereas the mean birth weight for the normal weight babies was 3054 grams with a standard deviation of 424 grams. Mean difference in birth weight between an LBW baby and a normal baby was found to be significant (843.24 gm; *p* < 0.0001, 95% CI: 745.94–940.55). 

### 3.2. Maternal Factors and Utilization of Antenatal Care Services

Table 1 summarizes the maternal characteristics. 402 mother-child pairs were analyzed and the majority of mothers were 20–34 years old (69.2%), upper caste (61.2%), and had a primary or greater education level (75.2%). Almost a half (49.8%) worked in the service/business/household sectors, the majority (65.6%) had second and third terciles of family income, most maternal in-laws/husbands were family heads (77.6%), slightly more than half (51.5%) were from the MATRI-SUMAN intervention area, the majority were from the Terai origin by birth (71.6%), non-vegetarian (79.6%), multiparous (60.9%), slightly more than half ( 52.5%) gave birth to a female baby and had four or fewer family members (52.7%), just over one-third (39.6%) had domestic animals, and the majority (66.4%) had a kitchen garden. Maternal factors, such as, caste/ethnicity, educational status, occupation, family income, head of the family, area of residence, parity, number of family members in a household, adequacy of living rooms for the family, and use of a kitchen garden were found to be significantly associated with LBW (Table 1).

Regarding ANC service utilization, more than half (59.7%) visited four or more ANCs, 58.5% consumed recommended doses of iron and folic acid tablets, 81.6% were properly immunized for tetanus and diphtheria, 81.3% consumed de-worming tablets, 84.3% got adequate rest and sleep, and about one in four received additional food during pregnancy. Utilization of the antenatal care services during pregnancy (as indicated by the number of antenatal care visits), consumption of recommended doses of iron and folic acid (IFA), immunization for tetanus and diphtheria, consumption of deworming tablets, adequate rest and sleep, and additional food intake during pregnancy, were significantly associated with LBW (Table 2).

### 3.3. Associations between Low Birth Weight and Maternal Factors and the Utilization of Antenatal Care Services

Table 3 shows the results of multivariate analyses on the determinants of LBW. Statistically significant factors that influenced LBW are listed in Table 1 and Table 2, and these were included in the multiple logistic regression model. Maternal socio-demographic factors, such as caste/ethnicity, educational status, occupation, family size, and other factors, such as sex of child, having access to a kitchen garden, and living in the MATRI-SUMAN intervention area were significantly associated with LBW. Similarly, the utilization of antenatal care services as indicated by number of ANC visits, taking recommended doses of IFA tablets and de-worming tablets, and additional food intake during pregnancy, were significantly associated with LBW.

Mothers from the Dalit caste/ethnicity group were four-times more likely (aOR 4.2; 95% CI (1.7–10.4)) to have an LBW baby than mothers from the Adibasi/Janajati and upper-caste groups. Similarly, illiterate mothers were eight-times more likely (aOR 8.1; 95% CI (2.9–22.4)) to have an LBW baby than literate mothers. Mothers that performed manual work were at greater risk (aOR 5.9; 95% CI (1.6–21.1)) of having an LBW baby than mothers with an agricultural or service/business/household occupation. Female children were twice more likely (aOR 2.0; 95% CI (1.0–4.1)) to be born with an LBW than male children. Mothers who had more than four family members had higher odds of having LBW babies (aOR 5.6; 95% CI (2.3–13.5)) than mothers who had four or fewer number of family members. Mothers in the MATRI-SUMAN intervention area were less likely (aOR 0.37; 95% CI (0.16–0.83)) to have an LBW baby than mothers not in the intervention area. Access to a kitchen garden reduced the risk (aOR 0.15; 95% CI (0.06–0.37)) of having an LBW baby. 

Mothers who had not visited ANCs were five-times more likely (aOR 5.1; 95% CI (1.1–22.6)) to have an LBW baby, and mothers who visited an ANC, less than four times, were three-times more likely (aOR 3.4; 95% CI (1.1–10.2)) to have an LBW baby than the mothers who visited four or more times. Not taking IFA tablets during pregnancy increased the risk (aOR 3.0; 95% CI (1.1–8.2)) of an LBW baby. Mothers who did not consume de-worming tablets during pregnancy were three-times more likely (aOR 3.1; 95% CI (1.0–13.8)) to have an LBW baby. Finally, not taking additional food during pregnancy was found to increase the risk (aOR 3.6; 95% CI (1.3–9.4)) of delivering an LBW baby. 

## 4. Discussion

We found a wide range of maternal factors, that is, the Dalit caste/ethnicity, illiteracy, occupation as a manual laborer, having four or more family members, and birth of a female child were significantly positively associated with an LBW, and that those with a kitchen garden and those who resided in the intervention area of the MATRI-SUMAN trial area were less likely to have LBW babies. In addition, lack of utilization of antenatal care services (as indicated by no ANC visit), fewer than four ANC visits, not consuming the recommended doses of IFA and de-worming tablets, and not consuming additional food during pregnancy, increased the risk of an LBW baby.

Our study revealed that babies born to mothers from the Dalit caste/ethnic group were at a greater risk of having an LBW baby than those from the Adibasi/Janajati and upper caste/ethnic group. Several previous studies have also observed relations between ethnicity and LBW [26,27,28]. The Dalit ethnic group in Nepal are one of the most deprived and marginalized, many lack basic amenities, and fail to utilize available maternity care services [25,29]. Results from previous studies have consistently demonstrated that Dalit women are at risk of having LBW babies in Nepal [30,31]. Like many other studies [17,18,32,33], we also found that illiterate mothers were more likely to give birth to LBW babies, which may be due to a lesser use of antenatal care services because of a lack of knowledge and decision-making power [34]. Lower maternal education has been previously reported to be associated with poor utilization of prenatal care services and low nutritious food intake, and thus, to increase the risk of LBW babies [35,36]. Additionally, adolescent marriage and pregnancy is one of the major causes of LBW babies and is prevalent among illiterate women [37]. Increasing girls’ education levels, female empowerment, raising health awareness through mass media, and nutritional counseling during antenatal check-ups, would help a lot to address the long-standing problem of LBW, in Nepal [18,34,38].

In the present study, maternal occupation as a manual laborer, four or more family members in a household, and birth of female child were found to increase the risk of LBW, while access to a kitchen garden or residing in the MATRI-SUMAN intervention area reduced the risk of LBW. Furthermore, we found that physical hard work was found to increase the risk of LBW babies, which concurred with the findings of two Indian studies and one Nepalese studies, which showed hard physical work during pregnancy increased the risk of LBW babies [39,40,41]. In another study, it was suggested that higher energy expenditure might lower maternal nutrients and adversely impact birth weight [42]. Incremental risks of LBW have also been attributed to maternal socioeconomic and demographic factors [17,43], possibly because socioeconomic conditions influence the use of essential maternal and child health services [44], maternal nutrition [45], and maternal decision-making power [46]. Our observation that female babies are more at risk of an LBW is supported by studies conducted in Kenya [47], Ethiopia [48,49], Nepal [20] and Australia [50]. 

Previous study reported male babies are less likely to have LBW, compared to their female counterparts because the male chromosome confers advantages in the determination of birth weight [51]. Interestingly, having access to a kitchen garden at home provides opportunities to increase dietary diversity and consume green leafy vegetables during pregnancy [52]. This study also revealed that mothers in the intervention area were found to be at a lower risk of having an LBW baby, indicating the beneficial effects of MATRI-SUMAN intervention [23] in lowering LBW cases. 

We also found that a lack of utilization of essential prenatal care services was linked to LBW. A number of previous studies are in agreement with our finding of a positive association between LBW and poor utilization of prenatal care services, as indicated by low numbers of ANC visits [18,20,32,33,53], non-consumption of IFA [18,20,32] and de-worming tablets [54,55], and no additional food intake [56,57] during pregnancy. An adequate number of antenatal visits aids the early recognition of pregnancy complications and promotes maternal nutrition and necessary care during pregnancy [58,59]. For example, an adequate consumption of IFA tablets reduces maternal anemia, the risks of pre-term birth and LBW [60], the consumption of de-worming tablets during pregnancy prevents worm infestation, and thereby, the proportion of LBW babies [54,61], and an extra meal taken during pregnancy ensures that the growing demand for calories during pregnancy, is met [62].

Previous studies have reported that mothers employed in the agricultural sector [63] have no maternal autonomy [3,46], are multi-parous [64], reside in a single-room apartment [65], have insufficient rest during pregnancy [66] and are at higher risk of giving birth to LBW babies. The univariate analysis conducted in the present study also demonstrated that mothers working in these sectors had a family income in the second tercile, an in-law/husband as the head of family, were multiparous, had an inadequate number of rooms in their homes, and had inadequate time for rest and sleep, were at higher risk of having an LBW baby. However, these associations disappeared after controlling for potential confounders in the multivariate regression analysis. 

The present study was conducted to examine an important child healthcare problem faced by many developing countries and had a high response rate. However, our findings should be understood in the light of its pitfalls. First, data collection was conducted in a cross-sectional manner, which prevented investigation of causal relationships. Second, information on socioeconomic and maternal factors was self-reported, and thus, might be subject to bias. Third, although calibrated instruments were used, the different instruments were used to measure the facility-born and home-born (newborn) babies, and this may have influenced their birth weights. Fourth, this study was conducted in a small rural area of Nepal on a relatively small cohort, so special precautions should be taken to generalize our study findings. Nonetheless, a number of clusters from a rural community of Nepal was enrolled in the study, which we feel has policy implications.

## 5. Conclusions

Child survival policies and programs in Nepal aimed at improving child health outcomes should pay more attention to maternal and antenatal care service utilization factors. In our opinion bespoke maternal and child health programs, such as MATRI-SUMAN, a capacity-building and text-messaging intervention designed to improve maternal and child health outcomes, should be promoted. We suggest future studies to explore the determinants of small-for-gestational-age and pre-term births, and the economic feasibility of MATRI-SUMAN interventions in Nepal and other south Asian countries.

## Figures and Tables

**Table 1:** Association between maternal factors and low birth weight (LBW) *.

Variables	*n* = 402 (%)	Low Birth Weight		*p*-Value
		Yes, *n* = 78 (%)	No, *n* = 324 (%)	
Age				
<20 year	91 (22.6)	20 (25.6)	71 (21.9)	0.674
20-34 year	278 (69.2)	53 (67.9)	225 (69.5)	
≥35 years	33 (8.2)	5 (6.4)	28 (8.6)	
Caste/ethnicity				
Upper caste group	246 (61.2)	27 (34.6)	219 (67.6)	<0.0001
Adibasi/Janajati	89 (22.1)	19 (24.4)	70 (21.6)	
Dalit	67 (16.7)	32 (41.0)	35 (10.8)	
Educational status				
Illiterate	100 (24.8)	46 (59.0)	54 (16.7)	<0.0001
Primary	145 (36.1)	21 (26.9)	124 (38.3)	
Secondary and above	157 (39.1)	11 (14.1)	146 (45.0)	
Occupation				
Labor	75 (18.7)	36 (46.2)	39 (12.0)	<0.0001
Agricultural work	127 (31.6)	24 (30.8)	103 (31.8)	
Service/business/HH works	200 (49.8)	18 (23.0)	182 (56.2)	
Family income				
1st tercile	138 (34.3)	39 (50.0)	99 (30.5)	<0.0001
2nd tercile	128 (31.8)	26 (33.3)	102 (31.5)	
3rd tercile	136 (33.8)	13 (16.7)	123 (38.0)	
Head of family				
Herself	90 (22.4)	10 (12.8)	80 (24.7)	0.024
Others (In-laws/ Husband)	312 (77.6)	68 (87.2)	244 (75.3)	
Resided in MATRI-SUMAN intervention area				
Yes	207 (51.5)	31 (39.7)	176 (54.3)	0.021
No	195 (48.5)	47 (60.3)	148 (45.7)	
Origin of residence				
Terai	288 (71.6)	51 (65.4)	237 (73.1)	0.172
Hill	114 (28.4)	27 (34.6)	87 (26.9)	
Dietary habit				
Non-vegetarian	320 (79.6)	63 (80.8)	257 (79.3)	0.776
Vegetarian	82 (20.4)	15 (19.2)	67 (20.7)	
Parity				
Primi	157 (39.1)	22 (28.2)	135 (41.7)	0.029
Multi	245 (60.9)	56 (71.8)	189 (58.3)	
Sex of Child				
Male	191 (47.5)	28 (35.9)	163 (50.3)	0.022
Female	211 (52.5)	50 (64.1)	161 (49.7)	
Family size				
4 and less person	212 (52.7)	12 (15.4)	200 (61.7)	<0.0001
>4 persons	190 (47.3)	66 (84.6)	124 (38.3)	
Living room in family				
Insufficient	176 (43.8)	57 (73.1)	119 (36.7)	<0.0001
Sufficient	226 (56.2)	21 (26.9)	205 (63.3)	
Domestic animals				
Yes	159 (39.6)	25 (32.1)	134 (41.4)	0.131
No	243 (60.4)	53 (67.9)	190 (58.6)	
Kitchen garden				
Yes	267 (66.4)	19 (24.4)	248 (76.5)	<0.0001
No	135 (33.6)	59 (75.6)	76 (23.5)	

* Chi-square test was applied and *p*-values < 0.05 were considered statistically significant.

**Table 2:** Association between the utilization of selected antenatal care services and low birth weight (LBW) *.

Variables	*n* = 402 (%)	Low Birth Weight		*p*-Value
		Yes, *n* = 78 (%)	No, *n* = 324 (%)	
ANC visit-end				
No	35 (8.7)	14 (17.9)	21 (6.5)	0.002
<4ANC	127 (31.6)	29 (37.2)	98 (30.2)	
4 or More	240 (59.7)	35 (44.9)	205 (63.3)	
Consumption of recommended dose of Iron and folic acid (IFA)				
Yes	235 (58.5)	34 (43.6)	201 (62.0)	0.003
No	167 (41.5)	44 (56.4)	123 (38.0)	
Immunized with recommended dose of Tetanus and diphtheria (TD)				
Yes	328 (81.6)	48 (61.6)	280 (86.4)	<0.0001
No	74 (18.4)	30 (38.4)	44 (13.6)	
Consumed de-worming tablet				
Yes	327 (81.3)	47 (60.3)	280 (86.4)	<0.0001
No	75 (18.7)	31 (39.7)	44 (13.6)	
Adequate rest and sleep taken				
SYes	339 (84.3)	59 (75.6)	280 (86.4)	0.019
No	63 (15.7)	19 (24.4)	44 (13.6)	
Additional food intake				
Yes	99 (24.6)	47 (60.3)	52 (16.0)	<0.0001
No	303 (75.4)	31 (39.7)	272 (84.0)	

* Chi-square test was applied and *p*-values of <0.05 were considered statistically significant.

**Table 3:** Relations between low birth weight (LBW) and maternal factors and the utilization of antenatal care services during pregnancy, by logistic regression analysis *.

Variables	OR (95% CI)	*p*-Value	aOR (95% CI)	*p*-Value
Maternal factors				
Intervention area				
Yes	0.55 (0.34–1.0)	0.022	0.37 (0.16–0.83)	0.009
No	1.0 (ref.)		1.0 (ref.)	
Caste/ethnicity				
Dalit	7.4 (3.9–13.8)	0.0001	4.2 (1.7–10.4)	0.0001
Adibasi/Janajati	2.2 (1.1–4.2)	0.0001	1.8 (0.8–5.1)	0.216
Upper caste group	1.0 (ref.)	-	1.0 (ref.)	-
Educational status				
Illiterate	11.3 (5.4–23.4)	0.0001	8.1 (2.9–22.4)	0.0001
Primary	2.2 (1.0–4.8)	0.0001	1.6 (0.9–8.6)	0.217
Secondary and above	1.0 (ref.)	-	1.0 (ref.)	-
Occupation				
Labor	9.3 (4.8–18.1)	0.001	5.9 (1.6–21.1)	0.006
Agricultural work	2.3 (1.2–4.5)	0.011	1.7 (0.5–5.3)	0.332
Service/ business/ HH works	1.0 (ref.)	-	1.0 (ref.)	-
Family income				
1st tercile	3.7 (1.8–7.3)	0.0001	1.1 (0.5–2.9)	0.761
2nd tercile	2.4 (1.1–4.9)	0.016	1.4 (0.3–1.9)	0.618
3rd tercile	1.0 (ref.)	-	1.0 (ref.)	-
Head of family				
Others (In-laws/ Husband)	2.2 (1.0–4.5)	0.027	1.9 (0.6–5.5)	0.206
Herself	1.0 (ref.)	-	1.0 (ref.)	-
Parity				
Multi	1.8 (1.0–3.1)	0.030	1.1 (0.5–2.4)	0.758
Primi	1.0 (ref.)	-	1.0 (ref.)	-
Sex of child				
Female	1.8 (1.0–3.0)	0.023	2.0 (1.0–4.1)	0.047
Male	1.0 (ref.)	-	1.0 (ref.)	-
Family size				
>4 persons	8.8 (4.6–17.0)	<0.0001	5.6 (2.3–13.5)	<0.0001
4 and less person	1.0 (ref.)	-	1.0 (ref.)	-
Living room in family				
Insufficient	5.0 (2.9–8.8)	<0.0001	1.2 (0.4–3.5)	0.664
Sufficient	1.0 (ref.)	-	1.0 (ref.)	-
Kitchen garden				
Yes	0.09 (0.05–0.17)	<0.0001	0.15 (0.06–0.37)	<0.0001
No	1.0 (ref.)	-	1.0 (ref.)	-
Utilization of antenatal care services during pregnancy				
ANC visit				
No	3.9 (1.8–8.3)	<0.0001	5.1 (1.1–22.6)	0.029
<4ANC	1.7 (1.0–3.0)	<0.0010	3.4 (1.1–10.2)	0.027
4 and more ANC	1.0 (ref.)	-	1.0 (ref.)	-
Consumption of recommended dose of Iron and folic acid (IFA)				
No	2.1 (1.2–3.4)	0.003	3.0 (1.1–8.2)	0.025
Yes	1.0 (ref.)	-	1.0 (ref.)	-
Immunized with recommended dose of Tetanus and diphtheria (TD)				
No	3.9 (2.2–6.9)	0.0001	2.2 (0.5–10.0)	0.295
Yes	1.0 (ref.)	-	1.0 (ref.)	-
Consumed de-worming tablet				
No	4.1 (2.4–7.3)	0.0001	3.1 (1.0–13.8)	0.049
Yes	1.0 (ref.)	-	1.0 (ref.)	-
Adequate rest and sleep taken				
No	2.0 (1.1–3.7)	0.020	1.5 (0.5–4.8)	0.412
Yes	1.0 (ref.)	-	1.0 (ref.)	-
Additional food intake				
No	7.9 (4.6–13.6)	0.0001	3.6 (1.3–9.4)	0.008
Yes	1.0 (ref.)	-	1.0 (ref.)	-

* All variables having *p*-value ≤ 0.1 were entered in a final multivariate logistic regression model and the statistical significance was considered for *p*-value < 0.05. Ref.: reference.
